# Allobetulin and Its Derivatives: Synthesis and Biological Activity

**DOI:** 10.3390/molecules16032443

**Published:** 2011-03-14

**Authors:** Wim Dehaen, Anastassiya A. Mashentseva, Talgat S. Seitembetov

**Affiliations:** 1 Molecular Design and Synthesis, Department of Chemistry, University of Leuven, Celestijnenlaan 200F, 3-3001 Leuven, Belgium; 2 Department of Chemistry, The L.N.Gumilev Eurasian National University, Munaitpasov str. 5, 010008 Astana, Kazakhstan; E-Mail: mashentseva.a@gmail.com; 3 Department of Biochemistry, Medical University “Astana”, Beybetshilyk. 51, 010000 Astana, Kazakhstan; E-Mail: samankul46@mail.ru

**Keywords:** triterpene, allobetulin, rearrangement, biological activity

## Abstract

This review covers the chemistry of allobetulin analogs, including their formation by rearrangement from betulin derivatives, their further derivatisation, their fusion with heterocyclic rings, and any further rearrangements of allobetulin compounds including ring opening, ring contraction and ring expansion reactions. In the last part, the most important biological activities of allobetulin derivatives are listed. One hundred and fifteen references are cited and the relevant literature is covered, starting in 1922 up to the end of 2010.

## 1. Introduction

Triterpenes and triterpenoids are numerous and widely distributed in Nature. Biosynthetically, they are derived from squalene. Earlier studies have focused on the isolation and structural elucidation of the compounds, and there is still a lot of ongoing research in this area that has been regularly reviewed by Connolly and Hill [1]. During recent years, several interesting biological properties were found for this class of compounds, which in combination with their low toxicities lead to an increased research effort [2,3]. More particularly, the oleanane group displays a number of significant pharmacological activities. Allobetulin (**2**) and its derivatives, obtained from the readily available lupane betulin (**1**), form a part of the oleanane group.

In this review, we summarize the chemistry of allobetulin analogs including: (1) their formation by rearrangement from betulin derivatives, (2) their further derivatisation, (3) their fusion with heterocyclic rings, and (4) the further rearrangements of allobetulin including ring opening, ring contraction and ring expansion reactions. In the final part (5), the most important biological activities of the allobetulin derivatives mentioned in sections 1–4 are listed. 

There are also a number of allobetulin derivatives that are isolated from plant extracts. For a recent example see [4]. These will not be treated in this review. We also did not cover the chemistry of the ring contracted or *seco*-derivatives of allobetulin, other than their formation from allobetulin derivatives.

## 2. Betulin-Allobetulin Rearrangement

**Scheme 1:**
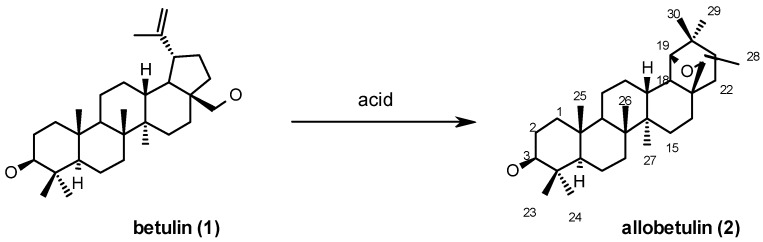
Rearrangement of betulin (**1**) to allobetulin (**2**).

In 1922, Schulze and Pieroh reported that when betulin (**1**) was heated in formic acid, an unexpected formate ester product resulted, that gave an isomeric product after saponification that was named allobetulin (**2**) (Scheme 1) [5]. At that time, very little was known about the structure of (allo)-betulin due to the lack of adequate characterisation techniques, but the authors were able to conclude that the obtained product was a monoalcohol, containing an ether function and an otherwise strongly rearranged structure as compared to the dialcohol betulin (**1**). Dischendorfer *et al.* determined the correct molecular *bruto* formula of **2** not much later [6]. In the following years several authors carried out similar rearrangements and prepared derivatives of allobetulin (**2**), but breakthroughs regarding its structure came only after the work of Davy [7] who oxidized the acetate of allobetulin to the corresponding 28-oxo derivative, and then saponified it to the alcohol and oxidized this compound to oxyallobetulone (**3**). The latter was identical to a product (“ketone-lactone-A”) derived by rearrangement of betulonic acid. Only recently was an X-ray structure of allobetulin (**2**) reported [8].

Various acidic conditions have been applied for this transformation, which is now known to belong to the class of Wagner-Meerwein rearrangements. Hydrobromic acid in chloroform [9], sulfuric acid in acetic acid [10], concentrated hydrochloric acid in ethanol [11,12] and even the highly toxic dimethyl sulfate [13] have been used for the transformation of **1** to **2** in moderate to good yields. The yield can be substantially improved by using acid reagents adsorbed on solid supports. Li *et al.* used “solid acids” such as sulfuric acid or tosic acid on silica, Montmorillonite K10 and KSF, bleaching clays and kaolinite to obtain allobetulin and its derivatives in close to quantitative yield [14]. Pichette *et al.* have used ferric nitrate or ferric chloride absorbed on silica gel or alumina to convert betulin (**1**) into allobetulin (**2**) in excellent yield. Longer reaction times lead to the formation of allobetulone (**4**) or A-ring contracted products, respectively [15]. Ferric chloride hydrate itself (not supported) was also used for a larger scale reaction (approx. 5 g, 92% yield) [16]. Trifluoroacetic acid [17] or bismuth triflate (*via* triflic acid liberated by hydrolysis) [18] also give excellent results for this transformation. Russian researchers, including patent literature, mention the use of diluted sulfuric acid [19] and orthophosphoric acid [20] to combine the process of extraction of **1** from birch bark and rearrangement to **2**. This rearrangement can in fact be seen as an interesting undergraduate laboratory experiment [21].

**Figure 1:**
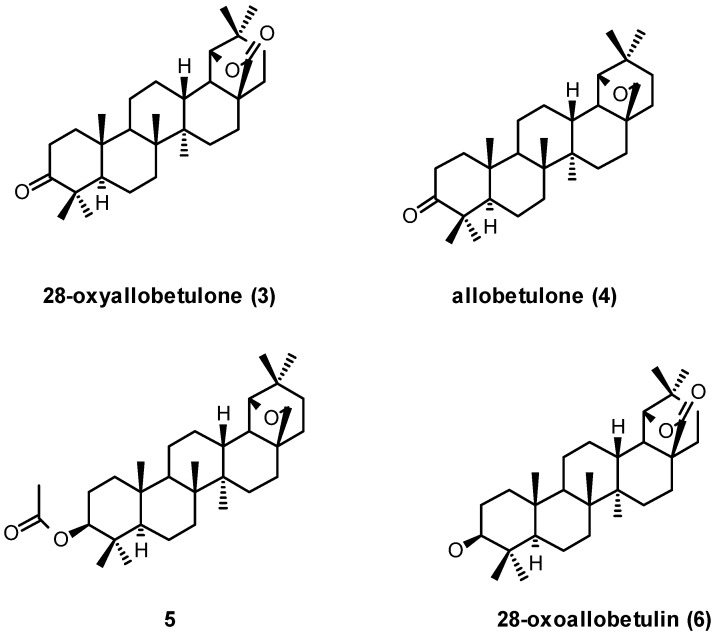
Structures of triterpenes **3-6**.

Simple derivatives of betulin, such as betulone, 3-acetylbetulin, and betulinic acid have been transformed by the above methods to the corresponding allobetulin analogs allobetulone (**4**), 3-acetoxyallobetulin (**5**), and 28-oxoallobetulin (**6**). Betulinic acid is slower to rearrange in comparison to other betulin analogs and may give substantial amounts of side products. 28-Oxoallobetulin (**6**) may be prepared more effectively in two steps by rearrangement of the 3-acetylated betulinic acid, followed by hydrolysis [22]. As mentioned earlier, rearrangement of betulonic acid or its methyl ester [23] affords triterpene **3**, which can be reduced back to **6** (Figure 1) [24].

**Figure 2:**
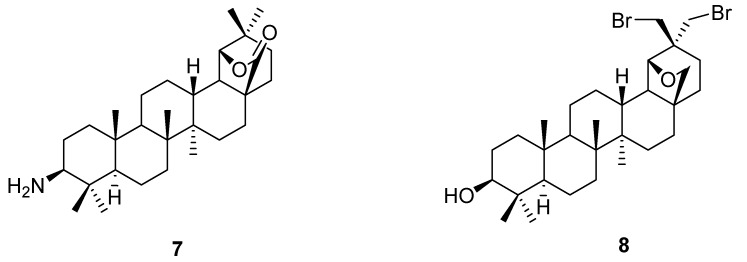
Structures **7** and **8**.

Another example is the preparation of 3-amino-28-oxoallobetulin (**7**) after attempted trifluoroacetic acid deprotection of the corresponding Boc-protected betulinic acid derivative [25]. Treatment of betulin (**1**) with bromine was reported to give a good yield of the dibromoallobetulin (**8**) [26]. The structure of rearrangement product **8** was proven by X-ray crystallography. However, this good yield is difficult to reproduce so an efficient procedure towards this interesting product is still lacking. Pradhan *et al.* likewise reported on the formation of the 3-acetylated 28-oxo analogs of **8** after treatment of the corresponding betulin derivative with *N*-bromosuccinimide in DMSO (Figure 2) [27,28,29]. 

**Scheme 2:**
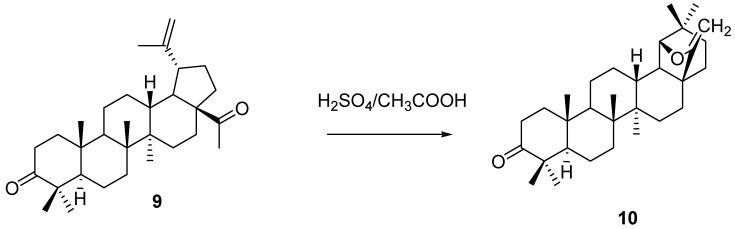
Rearrangement of ketone **9** to enol ether **10**.

Davy *et al.* prepared an interesting enol ether analog **10** of allobetulin via rearrangement (20% sulfuric acid in acetic acid) of the acetyl derivative of betulone (**9**). Ozonolysis of compound **10** afforded 28-oxoallobetulone (**3**), proving the enol ether structure (Scheme 2) [30].

**Scheme 3:**
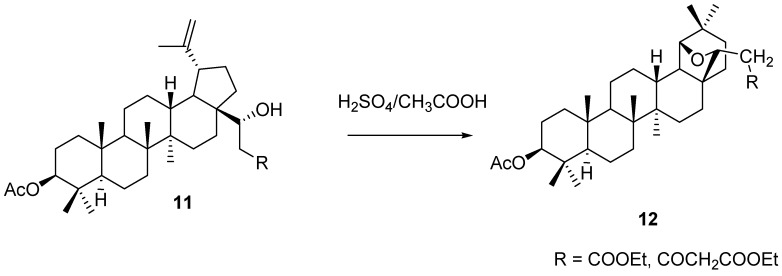
Rearrangement of alcohols **11** to allobetulin homologues **12**.

In the recent work of Czuk *et al.*, allobetulin homologues **12** were prepared in almost quantitative yields by trifluoroacetic acid induced rearrangement of secondary alcohols **11** that were prepared from 3-acetylated betulinic aldehyde by aldol condensation reactions (Scheme 3) [31].

**Figure 3:**
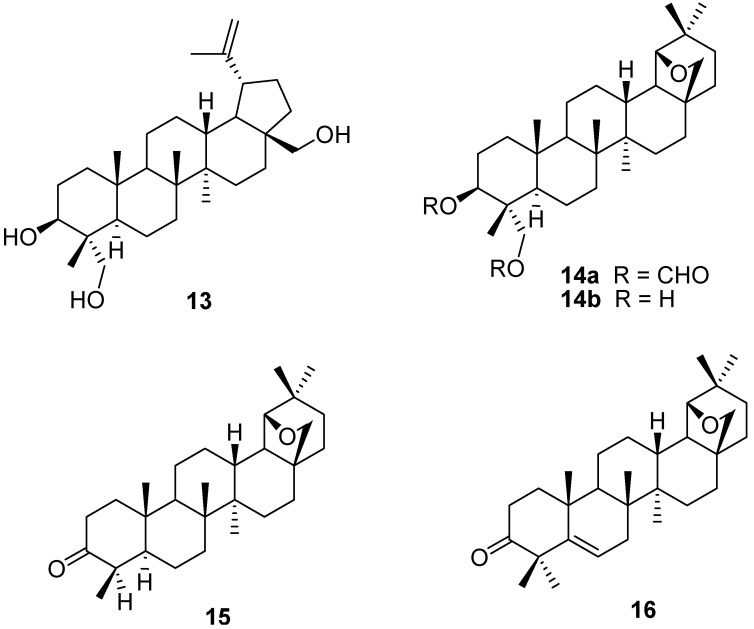
Structures of compounds **13-16**.

The naturally occurring 23-hydroxybetulin (**13**, obtained from the bark of *Sorbus aucuparia* L.) was transformed to the diformate **14a** (R = CHO) by an adaptation of the Schulze-Pieroh procedure [11]. Removal of the formate lead to 23-hydroxyallobetulin (**14b**, R = H). Oxidation of the latter with Jones reagent lead to formation of the norketone **15**, after decarboxylation of the intermediate ketoacid. The latter compound was used as a means to functionalize the B-ring, and 19β,28-epoxy-18α-olean-5-ene derivatives such as the interesting unsaturated allobetulone analog **16** were obtained after a bromination, dehydrobromination and methylation sequence (Figure 3) [32]. 

## 3. Simple Functionalisation Reactions of Allobetulin Analogs

Allobetulin (**2**) can be simply oxidized to the synthetically valuable allobetulone (**4**) by chromium(VI) reagents [2,33,34], Swern reaction [35] or sodium hypochlorite [36]. As mentioned previously, **4** can also be prepared in a one-pot procedure from betulin (**1**) [15]. 

**Figure 4:**
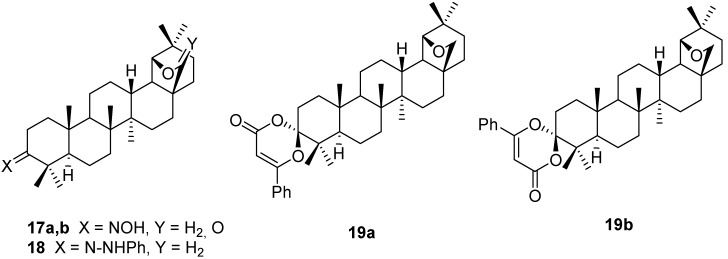
Structures of compounds **17-19**.

Allobetulone (**4**) was used to prepare the usual ketone analogues, such as the oxime **17a,b** (X = NOH, Y = H_2_ or O) [5,37], and the phenylhydrazone **18** (X = N-NHPh) [5]. Epimeric spiro compounds **19a,b** were obtained (as a 1:2 mixture) from **4** by hetero-Diels-Alder cycloaddition reaction with benzoyl ketene generated *in situ* from 5-phenyl-2,3-dihydrofuran-2,3-dione (Figure 4). The two isomers **19a,b** were isolated and characterized by X-ray crystallography [38]. The effect of the substituents on the cumulene and aryl fragments on the stereoselectivity was studied [39].

3-Acetoxyallobetulin (**5**) was oxidized to the lactone **6** with CrO_3_ in acetic acid [5,40], similarly **3** was prepared starting from allobetulone (**4**) [6]. Zhang *et al.* succeeded to oxidize allobetulin (**2**) directly to 28-oxoallobetulone (**3**, 87% yield), using sodium periodate/ruthenium trichloride as the reagent [41]. The selective reduction of lactone **3** to 28-oxoallobetulin (**6**) is another viable alternative to prepare this compound [24]. 

**Scheme 4:**
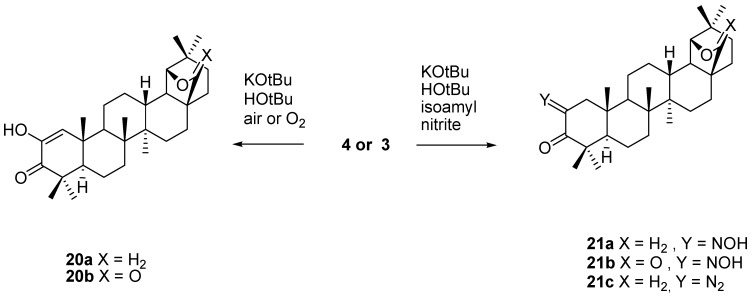
Oxidation and oximation of ketones **3,4**.

Base catalyzed oxidation of allobetulone (**4**) with oxygen as the reagent affords the 2-hydroxy enone derivative of allobetulin (**20**) [34,40,42,43]. Similarly, the oximes **21a,b** (X = H_2_, O) are prepared from **4** or **3** *via* condensation with isoamyl nitrite reagent [32]. Forster reaction of **21a** gave the corresponding 2-diazoallobetulone (**21c**, Scheme 4) [42].

**Figure 5:**
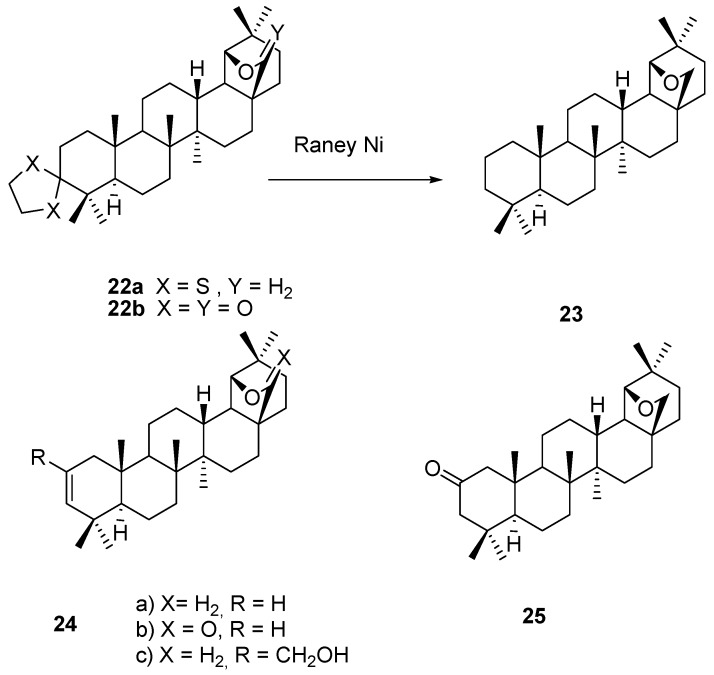
Structures of compounds **22-25**.

Ethylenedithioketal **22a** (X = S, Y = H_2_) was prepared from allobetulone (**4**) and reduced to allo-betulane (**23**) with Raney nickel [44]. The 28-oxyallobetulone ketal **22b** (X = Y = O) was prepared in 86% yield from triterpene **3** and ethylene glycol [41]. Other ketals were also prepared from allobetulone (**4**) [45]. Allobetulenes **24a,b** that are of importance as biomarkers were prepared from allobetulin (**2**, X = H_2_) or 28-oxoallobetulin (**6**, X = O) via tosylation in pyridine and elimination of toluenesulfonic acid [14]. The alkene **24a** is known from older work by the name of γ-allobetulin [46,47]. This alkene **24a** was subjected to the Prins reaction, leading to the alcohol **24c** (R = CH_2_OH) [48]. Interestingly, allobetulone (**4**) was isomerized to the 2-keto triterpene **25** in the presence of sulfur and morpholine (Figure 5) [49].

**Figure 6:**
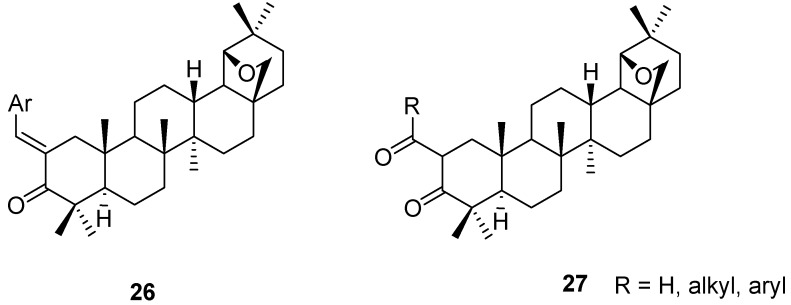
Structures of triterpenes **26** and **27**.

Aldol condensation reactions of allobetulone (**4**) with benzaldehydes and heterocyclic aldehydes lead to the α,β-unsaturated ketones **26** [6,50]. Similarly, Claisen condensations of **4** with formate and oxalic esters have been used to prepare the synthetically useful 1,3-diketones **27** or their enol tautomers (Figure 6) [16,51,52,53]. The formyl derivative **27** (R = H) was converted into the 2-fluoro-methylidene derivative by treatment with DAST [54].

**Figure 7:**
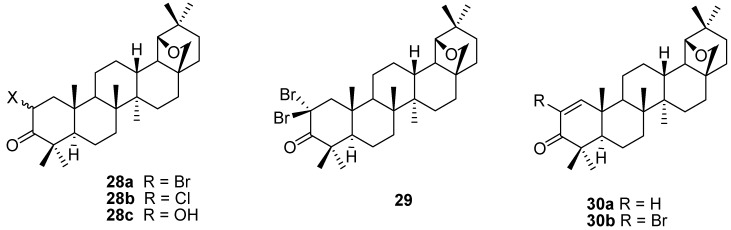
Structures of triterpenes **28-30**.

Both the 2-monobrominated (mixture of the 2α- and 2β-epimers) **28a,b** (X = Br) and the 2,2-dibrominated derivative **29** can be prepared by controlled reaction of allobetulone (**4**) with different brominating reagents [55,56,57,58,59,60]. The corresponding chlorinated derivatives **28a,b** (X = Cl) are also known [61]. The conformations of these brominated triterpene derivatives were studied in detail [58] and an X-ray structure of isolated 2β-bromoallobetulone (**28b**) was reported. [62] Dehydrobromination of **28** or **29** gave unsaturated ketones **30a,b** [55,59]. Ketone **30a** (R = H) was also prepared directly via phenylselenic anhydride oxidation of allobetulone **4** [39] and was used as a Michael acceptor for cyanide anion (Figure 7) [41].

**Figure 8:**
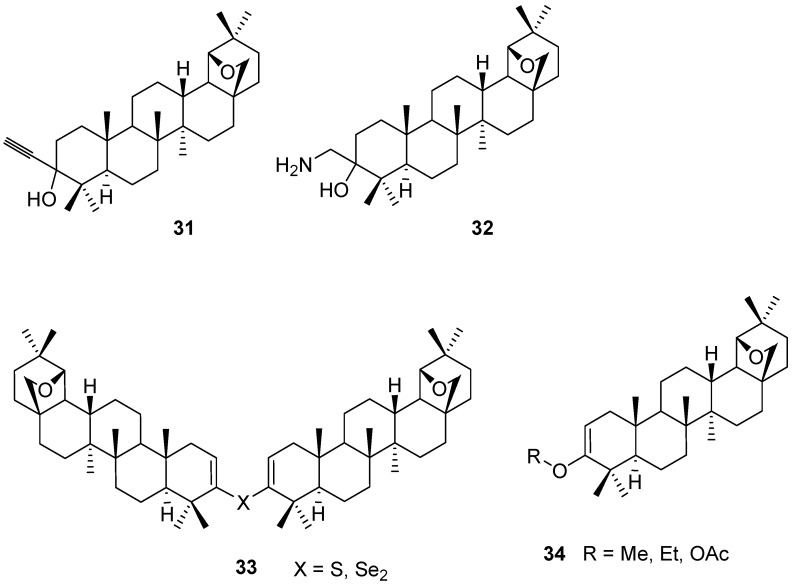
Structures of triterpenes **31-34**.

Addition of acetylide to allobetulone (**4**) affords a monoacetylene **31** [63]. Trimethylsilylcyanide addition to **4** followed by reduction yields aminoalcohol **32** [35]. A dimeric bis(allobetulenyl) sulfide **33** (X = S) is isolated after treatment of allobetulone (**4**) with Lawesson’s reagent [64] the corresponding diselenide **33** (X = Se_2_) was isolated after attempted dehydrogenation of **4** with selenium dioxide [59]. Enol acetates and ethers **34** were also prepared starting from allobetulone (**4**)(Figure 8) [65,66].

**Scheme 5:**
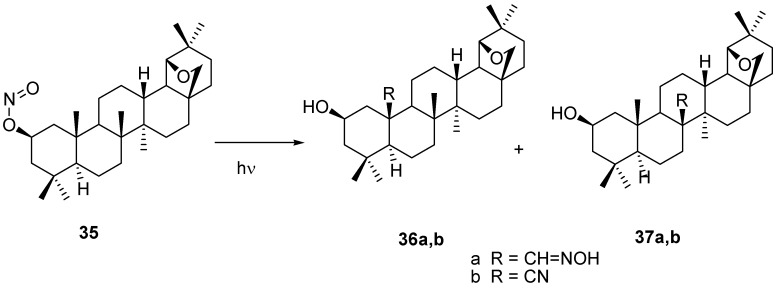
Photolysis of nitrite **35**.

A remarkable photolytic transformation (Barton reaction) of 2-nitrite **35**, derived from the ketone **25** after reduction and esterification with nitrosyl chloride, lead to the formation of two regioisomeric aldoximes **36** (40%) and **37** (40%) [67,68,69]. The remotely functionalized (C-25 and C-26) oximes **36a** and **37a** were further converted into nitriles **36b** and **37b** (Scheme 5).

Simple ester derivatives **38** of allobetulin (**2**) or its 28-oxo analog **6** may be prepared *via* acylation of the 3-βOH function [5,70,71]. Acylation with cyclic anhydrides leads to monoacidic ester derivatives with improved water solubilities [71]. Alternatively, R groups containing ionic functionalities (ammonium, sulfonate) or polyethylene glycol solubilizers are attached [72]. Next to acylation, sulfonylation and phosphorylation reactions were also described [71]. The oximes **17a,b** may also be used to prepare O-acylated derivatives **39** [37,73].

**Figure 9:**
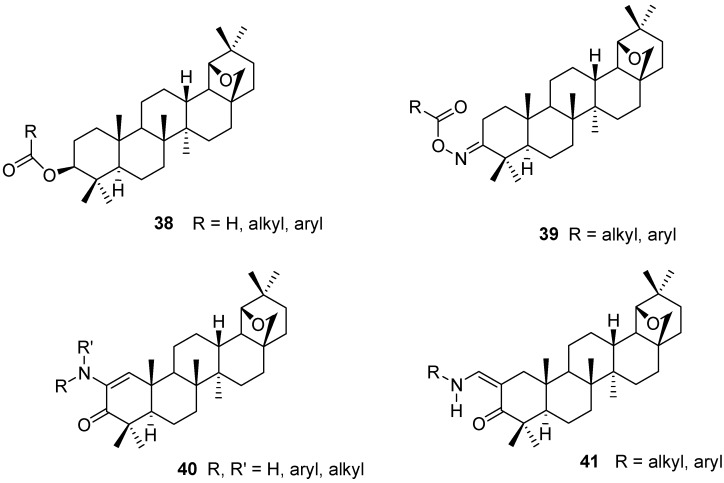
Structures of triterpenes **38-41**.

Enamines **40** are prepared from the enol acetate **34** (R = Ac) by epoxidation and condensation with primary or secondary amines. The reaction involves an oxidation, probably effected by adventitious oxygen [67]. 2-Alkylaminomethylene derivatives **41** of allobetulone (**4**) were prepared from the Claisen ester condensation product **27** (R = H) and primary amines (Figure 9) [53].

**Figure 10:**
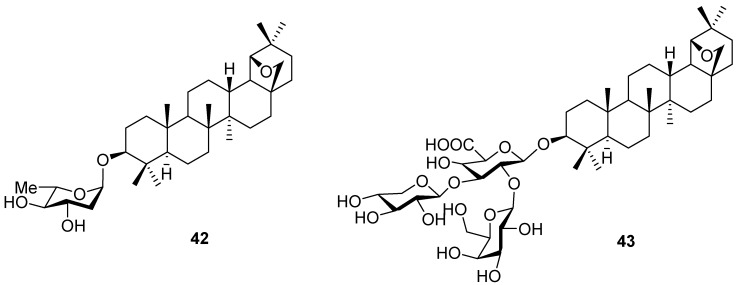
Structures of triterpene saponins **42** and **43**.

Glycosides and saponins with allobetulin (**2**) or its 28-oxo derivative **6** as aglycones were prepared by reacting the 3β-hydroxy function with sugar derivatives, such as glycals [74,75,76,77,78] or a large collection of trichloroacetimidates [22,79,80,81]. This modification, see for instance glycoside **42** [78], saponin **43** [79] and the glycoside derivative of **20a** [40], greatly enhances water solubility and hence influences the biological properties (Figure 10).

## 4. Ring Fusion to the A-Ring of Allobetulin

**Scheme 6:**
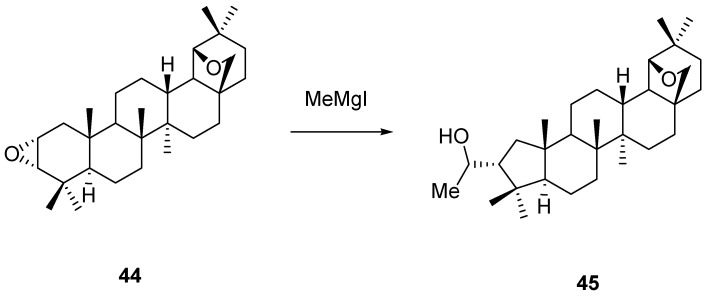
Ring opening of epoxide **44** with Grignard reagent to afford **45**.

2,3-Epoxides may be formed by ring closure of the corresponding bromohydrins, available from reduction of 2-bromoallobetulones **28** (X = Br), by epoxidation of alkene **24**a [59,82] or by oxidation reactions of enol acetate **34** (R = Ac) as mentioned above [66]. The main feature of these epoxides is their propensity for ring opening reactions with nucleophiles [52,61]. Interestingly, 2α,3α-epoxide **44** on treatment with a methyl Grignard reagent underwent rearrangement (see also next part 3) before addition of the organometal, affording nor-A alcohol derivative **45**. The 2β,3β-isomeric epoxide underwent a similar rearrangement/addition sequence (Scheme 6) [83].

**Figure 11:**
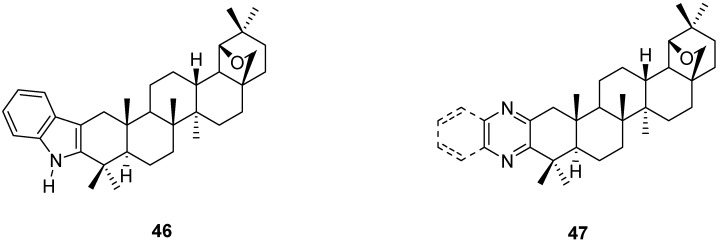
Structures of fused triterpenes **46** and **47**.

Allobetulone (**4**) and its substituted derivatives are the starting point for the annelation of allobetulin with heterocyclic rings. For instance, Fischer indole synthesis starting from arylhydrazine and ketone **4** gave the fused indole **46** [84,85]. 2-Hydroxyenone **20a** was condensed with diamines such as 1,2-diaminobenzene and 1,2-ethylenediamine to give the corresponding (benzo)pyrazine derivatives **47** (Figure 11) [43,45,49,86].

**Figure 12:**
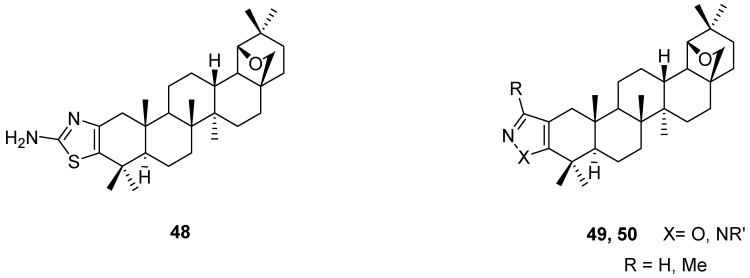
Structures of fused triterpenes **48-50**.

Hantzsch type synthesis starting from the α-bromoallobetulone (**28a**, R = Br) and thiourea gave an aminothiazolo fused triterpene **48** [87]. Condensation of the 1,3-dicarbonyl derivatives **27,** prepared by Claisen ester condensation of **4,** with hydrazine or hydroxylamine gave pyrazoles **49** or isoxazoles **50**, respectively [51]. In the case of alkylhydrazines two isomeric pyrazoles with [b] and [c] fusion are formed (Figure 12) [88].

## 5. Further Rearrangements of Allobetulin, Including Ring Contractions and Ring Expansions

**Figure 13:**
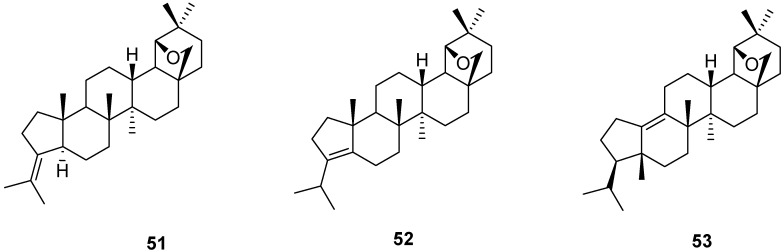
Structures of apoallobetulins **51-53**.

Often, the rearrangement of betulin (**1**) to allobetulin (**2**) is accompanied with the formation of the dehydrated, isomeric “apoallobetulins”. The latter have a variety of structures and can also be obtained from isolated allobetulin (**2**) by treatment with different acidic reagents. The structure of the δ-allobetulin **51** obtained by treatment of allobetulin (**2**) with PCl_5_ or phosphorous pentoxide at 0 °C [5,6,15] was shown later by ozonolysis to have an exocyclic double bond [89]. The so-called α-apoallobetulin **52** has an endocyclic double bond and is formed on treatment of betulin (**1**) with Fuller’s earth [6,89]. More recently, different solid acids such as Montmorrilonite K10 have successfully transformed **1**, **2** or even the δ–isomer **51** to mixtures of **52** and the “rearranged α-apoallobetulin” [47] **53**. In general, the amount of **53** in the mixture increases at higher temperatures. 28-Oxo derivatives of **52** and **53** are formed accordingly from betulinic acid or 28-oxoallobetulin (**6**) [15]. Silica- or alumina supported FeCl_3_ hydrate gave a similar mixture (55:45 ratio) of **52** and **53** on extended reaction of betulin (**1**), via allobetulin (**2**) [16]. The reaction of allobetulin (**2**) with PCl_5_ has been reinvestigated and was shown to lead directly to **52** at slightly higher temperatures (5–10 °C). At −10–0 °C, the expected **51** was formed [47]. The highest yields and selectivities of apoallobetulin isomers were obtained on treatment of betulin (**1**) with bismuth triflate. The relative amount of catalyst is important. Thus, heating **1** with 20 mol% catalyst for 40 h at reflux in dichloromethane gave 98% yield of **52**. On the other hand, heating of **1** or **52** for 8–15 h in the same solvent with 50 mol% bismuth triflate gave the isomer **53** almost quantitatively (96–98% yield) (Figure 13) [19].

**Figure 14:**
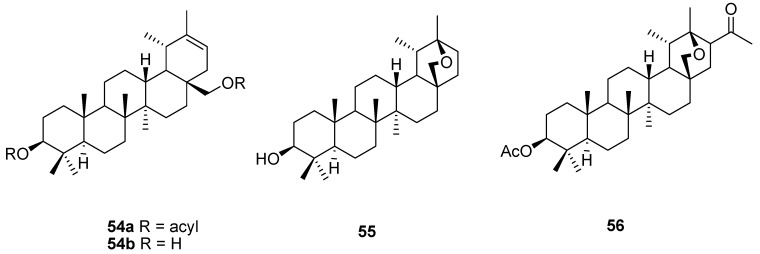
Structures of ring opened allobetulin derivatives **54-56**.

Treatment of allobetulin (**2**) with acid chlorides in high boiling solvents leads to rearranged and ring opened diacylated products **54a**, that can be saponified to the so-called “heterobetulin” **54b**, which has an ursane framework [9,47,90,91]. “Alloheterobetulin” **55** is a ring closed isomer of the latter which can be obtained after treatment of **54b** with toluenesulfonic acid [92]. A remarkable rearrangement/O,C-diacylation was recently reported to occur (55% yield of **56**) when allobetulin (**2**) was treated with acetic anhydride and a few drops of perchloric acid (Figure 14) [93].

**Figure 15:**
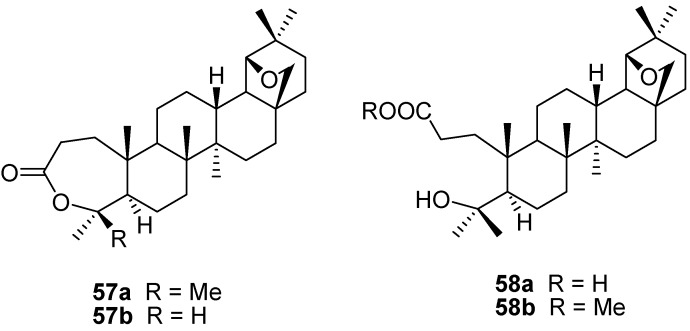
Structures of A-*seco* derivatives **57** and **58**.

The Baeyer-Villiger oxidation of allobetulone (**4**) was investigated by different groups under different circumstances [40,94,95]. With MCPBA in dichloromethane, the main product (83%) is the ring-expanded lactone **57a**. Other peracids (performic, peracetic) give similar results. However, reaction of **4** with MCPBA in the presence of acid (acetic + sulfuric) leads to the formation of a nor-lactone **57b**. The 3,4-*seco* derivatives **58a,b** were obtained in good yield either from **57a** by alkaline hydrolysis or directly from **4**, carrying out the oxidation in methanol with a trace amount of sulfuric acid [95]. Larger amounts of acid (0.15%) lead to the formation of the 2α-hydroxyallobetulone **28c** in good yield (86%) (Figure 15) [65].

**Figure 16:**
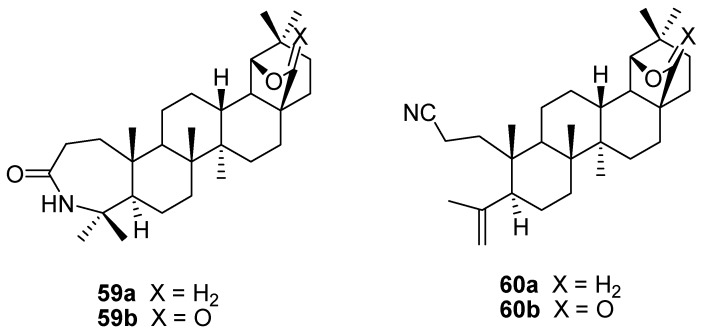
Structures of ring expanded and ring opened triterpenes **59** and **60**.

The Beckmann rearrangement of allobetulin oxime **17a**, induced by TsCl/pyridine or phosphoryl chloride, gave rise to the formation of a lactam **59a** (major product) and a 3,4-*seco*-triterpene nitrile **60** (minor product). The lactam **59a** could be transformed into the nitrile **60** on extended heating [96,97]. Upon Schmidt reaction of methyl betulonate or Beckmann rearrangement (POCl_3_) of its oxime, the 28-oxo derivatives **59b** and **60b** were formed after two consecutive rearrangements [98]. Other 2,3-*seco*-derivatives were prepared via Beckmann fragmentation of allobetulin derivatives (Figure 16) [33,45,99,100]. 

**Figure 17:**
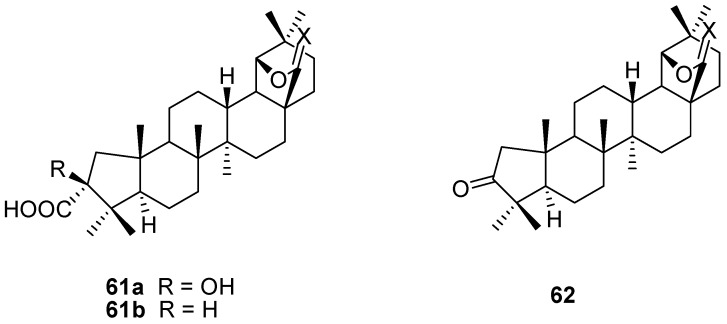
Structures of A-ring contracted triterpenes **61** and **62**.

The dibromoallobetulin **29** underwent a *quasi*-Favorskii rearrangement on treatment with base, leading to the ring contracted product **61a**. Oxidative decarboxylation of the latter with lead tetraacetate gave the norketone **62** [45,56,96,101]. The latter is an interesting starting material that was used in many follow-up reactions that will not be discussed here. Benzilic acid rearrangement of diketone **20a** gives the same hydroxyacid **61a**. The photochemical Wolff rearrangement of diazo compound **21c** gave the ring contracted carboxylic acid **61b** (Figure 17) [45].

**Scheme 7:**
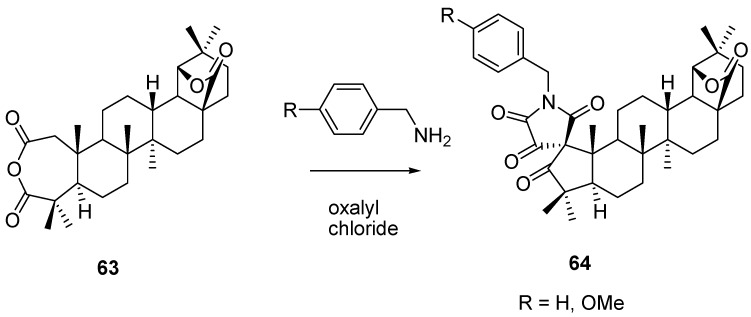
Spirocyclic triterpenes **64**.

Dischendorfer reported oxidation of the A-ring of 28-oxoallobetulone **3** to “allobetulinic acid” which formed a cyclic anhydride **63** [102,103]. This *seco*-derivative **63** was recently used to prepare spirocyclic derivatives **64** after treatment with benzylamines and oxalyl chloride [104]. Recently, the diacid analog of **63** was prepared by ozonolysis of the Claisen ester condensation product of **3** (*i.e.* the 3-oxo analog of **27**) [105]. This procedure has some similarity with earlier work by Ruzicka, who used chromic acid to prepare the diacid from hydroxymethyleneallobetulone (**27**, R = H) or directly from allobetulin (**2**) (Scheme 7) [106]. 

**Figure 18:**
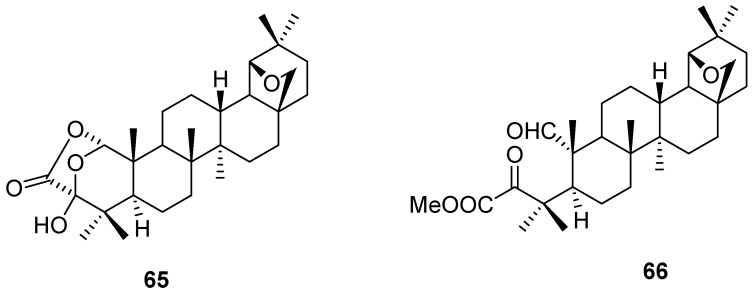
Structures of oxidized triterpenes **65** and **66**.

The nearly insoluble lactone **65** was formed in low yield (24%) on oxidation of allobetulone (**4**) with chromic acid. Treatment of **65** with diazomethane gave the 1,2-*seco* derivative **66** in good yield (Figure 18) [107].

**Figure 19:**
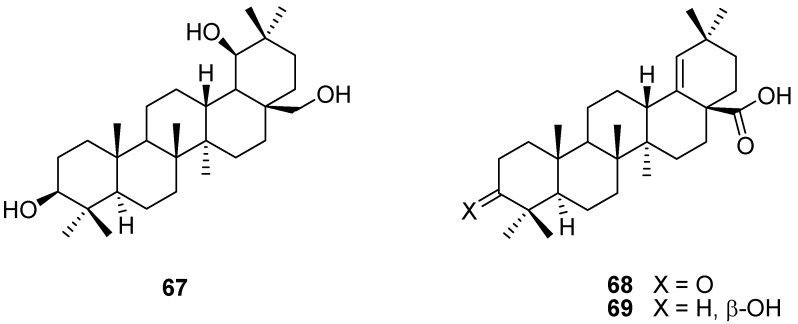
Structures of triterpenes **67-69**.

Another obvious position for ring cleavage is the lactone bridge of 28-oxoallobetulin derivatives. This bridge is quite stable towards saponification, but LiAlH_4_ reduction of the 3β-acetoxy derivative of **5** [108] or 28-oxoallobetulone (**3**) [25,30,109] gives a germanicanetriol derivative **67** which was further transformed to different germanicanes by selective acylation, oxidation and dehydration reactions [108,109]. The lactone ring of the protected 28-oxoallobetulone **22b** was reductive cleaved with LiAlH_4_ and after deprotection, 28-acetylation, dehydration with POCl_3_, saponification, and stepwise oxidation, moronic acid (**68**) and the reduced morolic acid **69** were obtained (Figure 19) [41]. In general, allobetulin and its derivatives are important starting materials for the synthesis of rare germanicanes and olean-18(19)-ene triterpenoids.

**Figure 20:**
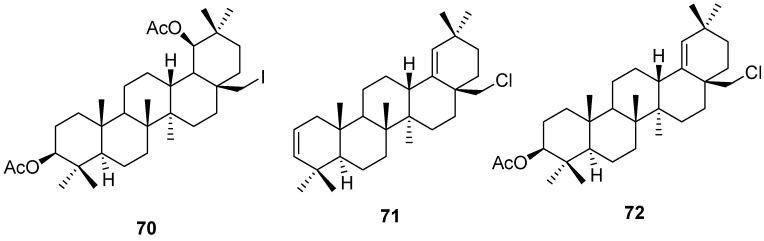
Structures of triterpenes **70-72**.

Treatment of allobetulin (**2**) with sodium iodide/acetyl chloride at reflux in acetonitrile lead to the formation of iodinated diacetate **70** [110]. Treatment of allobetulin (**2**) or its 3-acetate with POCl_3_ in refluxing pyridine similarly gave dialkene **71** or the corresponding acetate **72** (Figure 20) [16].

## 6. Biological Properties of Allobetulin Analogs

The biological properties of betulin, betulinic acid and its derivatives are well known [2] and often activity studies of allobetulin derivatives are found back in the literature together with or in comparison to their betulin isomers. A wide spectrum of biological properties have been reported, including antiviral, antifeedant, immunotropic, antibacterial, antifungal, and anti-inflammatory activities, cytotoxicity and inhibition of glycogen phosphorylase activities.

### 6.1. Antiviral properties

In 1995, it was found that allobetulin (**2**) itself showed moderate inhibitory activity against the influenza B virus [111]. It was claimed in the patent literature that different derivatives of allobetulin, including **2** and its 3-*O*-acylated and phosphorylated derivatives, **4**, **30a**, **32**, exhibited significant antiviral activity and could be used to treat herpes virus (HSV-herpes simplex virus) infection [35]. Also in 2002, compound **3** was shown in cell culture to inhibit influenza A growth while being inactive against HSV and the enterovirus ECHO-6 [112]. Somewhat later, allobetulin derivatives **3**, **6**, and different *O*-acylated oximes **39** were tested against several viruses such as HSV, influenza and ECHO-6 [24,37]. In fact, the non-acetylated oxime **17a** had the largest effect against influenza virus A, while being only moderately active against enterovirus ECHO-6 and inactive with respect to HSV. The *N*-acetylated oximes **39** had a moderate activity towards HSV, but were inactive against the other viruses [37]. It was confirmed that 28-oxoallobetulone (**3**) strongly inhibited the influenza virus, but did not influence HSV reproduction [24]. Rearranged product **56** showed only moderate inhibition of the *Papilloma* virus [93].

### 6.2. Antifeedant properties

In 1990, Lugemwa *et al.* reported high antifeedant activity against the bollworm larvae, *Heliothis zea*, for the glycoside derivative of **30a**. Simple allobetulin derivatives such as **2**, **20a** and **30a** itself were not active. The antifeedant property was selective and the glycoside did not display high activity against either the Colorado potato beetle (*Leptinotarsa decemlineata*) or the fall armyworm (*Spodoptera frugiperda*) [40].

### 6.3. Immunotropic activities

Different 2-substituted allobetulone derivatives, including the formyl analog **27** (R = H) and different condensation products with amines **41** were screened [53]. In fact, compounds **27** and **41** (R = ^i^Pr) had the most promising activity combined with low toxicity. In later work it was shown that these compounds had high biological activity on chronic administration and that their immunosuppressive activity was the result of toxic effect on the lymphocytes [113].

### 6.4. Antibacterial and antifungal activities

Compounds **2** and **3** were taken into a screening of 32 betulin derivatives against *Chlamydia pneumoniae*. Allobetulin (**2**) was equal to betulin (**1**) in antichlamydial activity (48%), but 28-oxoallobetulone (**3**) was inactive [114].

### 6.5. Anti-inflammatory and anti-ulcer properties

Biological tests on mice with the carrageenan and formalin edema models showed that acylated derivatives of allobetulin (**2**) possessed anti-inflammatory activity comparable to ortophen (diclofenac) [71,115]. Moderate antiulcer activity of **3** and 3-*O*-acylated allobetulin derivatives were observed in mice [112,115].

### 6.6. Cytotoxicity

The cytotoxicity of pyrazine and quinoxaline derivatives of allobetulin (**47**) was tested against a T-lymphoblastic leukemia cell line and found to be lower than the fused triterpene analogs based on unrearranged betulin or betulinic acid [86]. Pichette *et al.* did a study on the cytotocitity of betulin- and allobetulin-derived 3β-*O*-monodesmosidic saponins (such as **42**) with higher hydrosolubility and better pharmacokinetics. *In vitro* anticancer activity of saponins derived from **2** and **6** showed that the bioactivity for these glycosides was only moderate (IC_50_ 30–40 μM/L), as compared to the corresponding betulinic acid derivatives (IC_50_ 7.3–10.1 μM/L) [23]. The *in vitro* toxicity of **67** (human lung carcinoma or human colorectal adenocarcinoma assay) was comparable to that of betulinic acid or 5-fluorouracil [108]. Chacotrioside saponins such as **43** were fourfold superior to betulinic acid against human breast (MCF7) and prostate (PC-3) adenocarcimas cell lines. Moreover, chacotriosides bearing non-polar functions at the C-28 position had a haemolytic activity against red blood cells [80]. Allobetulin derivatives **12** with 28-functionality were reported by Czuk *et al.* to have moderate cytotoxicity [31].

### 6.7. Inhibition of glycogen phosphorylase

Morolic acid (**69**) (IC_50_ 70.3 μM/L), its 3-epimer (IC_50_ 34.5 μM/L) and 3-*O*-acetylated derivative (IC_50_ 32.7 μM/L) were shown to cause moderate inhibitory activity against rabbit muscle glycogen phosphorylase [41].

## 7. Conclusions

Allobetulin and its analogues are easily accessible starting from the corresponding betulin derivatives. Although a large structural variety of allobetulin analogs is already available by functionalisation, ring fusion to the A ring, further rearrangements, ring contractions, ring expansions, and ring cleavages, there is still much chemical space unexplored. Further investigations are certainly worthwile because of the interesting bioactivities.
